# Myeloid-derived suppressor cells-induced exhaustion of CD8 + T-cell participates in rejection after liver transplantation

**DOI:** 10.1038/s41419-024-06834-z

**Published:** 2024-07-16

**Authors:** Liu-Xin Zhou, Yi-Zhou Jiang, Xin-Qiang Li, Jin-Ming Zhang, Shi-Peng Li, Lin Wei, Hai-Ming Zhang, Guang-Peng Zhou, Xiao-Jie Chen, Li-Ying Sun, Zhi-Jun Zhu

**Affiliations:** 1 Liver Transplantation Center, National Clinical Research Center for Digestive Diseases, Beijing Friendship Hospital, Capital Medical University, Beijing, China; 2 State Key Lab of Digestive Health, Beijing, China; 3 Department of Critical Liver Diseases, Liver Research Center, Beijing Friendship Hospital, Capital Medical University, Beijing, China; 4 Organ Transplantation Center, Affiliated Hospital of Qingdao University, Qingdao, China; 5 Department of Hepatopancreaticobiliary Surgery, Henan Provincial People’s Hospital, Zhengzhou University, Zhengzhou, China

**Keywords:** Allotransplantation, Immune cell death

## Abstract

Liver transplantation (LT) rejection remains the most pervasive problem associated with this procedure, while the mechanism involved is still complicated and undefined. One promising solution may involve the use of myeloid-derived suppressor cells (MDSC). However, the immunological mechanisms underlying the effects of MDSC after LT remain unclear. This study is meant to clarify the role MDSCs play after liver transplantation. In this study, we collected liver tissue and peripheral blood mononuclear cells (PBMC) from LT patients showing varying degrees of rejection, as well as liver and spleen tissue samples from mice LT models. These samples were then analyzed using flow cytometry, immunohistochemistry and multiple immunofluorescence. M-MDSCs and CD8 + T-cells extracted from C57/BL6 mice were enriched and cocultured for in vitro experiments. Results, as obtained in both LT patients and LT mice model, revealed that the proportion and frequency of M-MDSC and PD-1 + T-cells increased significantly under conditions associated with a high degree of LT rejection. Within the LT rejection group, our immunofluorescence results showed that a close spatial contiguity was present between PD-1 + T-cells and M-MDSCs in these liver tissue samples and the proportion of CD84/PD-L1 double-positive M-MDSC was greater than that of G-MDSC. There was a positive correlation between the activity of CD84 and immunosuppressive function of M-MDSCs including PD-L1 expression and reactive oxygen species (ROS) production, as demonstrated in our in vitro model. M-MDSCs treated with CD84 protein were able to induce co-cultured CD8 + T-cells to express high levels of exhaustion markers. We found that CD84 regulated M-MDSC function via expression of PD-L1 through activation of the Akt/Stat3 pathway. These results suggest that the capacity for CD84 to regulate M-MDSC induction of CD8 + T-cell exhaustion may play a key role in LT rejection. Such findings provide important, new insights into the mechanisms of tolerance induction in LT.

## Introduction

Liver transplantation (LT) remains the most effective therapy for end-stage liver diseases. However, despite recent advances achieved in many aspects of the LT procedures [1], post-transplant graft rejection continues as the main obstacle affecting the long-term survival of these recipients [2]. Prolonged administration of immunosuppression drugs raises the risk of infection, impaired renal function, metabolic syndrome and the development of malignant tumors within other systems of the body, all of which may worsen the prognosis of LT recipients [3]. Accordingly, a better understanding of the underlying mechanisms of immunological rejection and means to alleviate this process represent an important, but challenging, area of investigation with regard to organ transplantation.

During the remodeling process that occurs after LT, the micro-immunological environment dramatically changes, as the relative number, rate, phenotype and functions of immune cells are markedly altered and eventually participate in the rejection response [4, 5]. T-cells play a crucial role in post-LT rejection through a series of immunological effects, including the identification of graft antigens, interactive responses with other immune cells and cytolysis effects [6]. Besides, CD8 + T-cells can directly induce acute rejection.

T-cells may manifest a declination or even failure in the activation, proliferation and secretion of cytokines in response to long-term exposure to antigens, an effect which is referred to as T-cell exhaustion [7]. These ‘exhausted’ T-cells then show a dampened immune response capacity to antigens [8], as well as diminished immunological surveillance effects and cytotoxicity [9].

Myeloid-derived suppressor cells (MDSC) are a group of cells consisting of immature mononuclear macrophages, dendritic cells (DC) and precursor granulocytes, all of which can exert immuno-suppressive effects [10]. MDSC, which can be divided into granulocyte-like (G-MDSC) or monocyte-like (M-MDSC) phenotype subpopulations, have been reported to participate in the pathophysiological processes of a number of diseases through their capacity to influence CD8 + T-cell functions [11, 12]. With T-cells serving as target cells, M-MDSC plays a crucial role in the immunoregulatory effects associated with organ transplantations [13]. Meanwhile, evidence begins to accumulate that Stat3 pathway was crucial for the immunosuppression function of MDSCs [14, 15], inhibiting Stat3 may be able to abrogate MDSC immunosuppressive capacity [16]. Most research involving MDSC has been mainly focused in the area of tumor immunology [17]. Results of past and recent studies have demonstrated the immunosuppressive efficacy of MDSC in heart transplant and mice skin, corneal transplant [18–21]. However, very little work have been directed at investigating the effects of MDSC in LT.

CD84 (aka Signaling lymphocyte activating molecule family member 5) is a member of the white cell membrane surface CD2 receptor immunoglobulin superfamily and has been reported to participate in the activation of T-cells and NK-cells [22, 23]. Recent evidence show that CD84 participates in the development and progression of chronic lymphocyte leukemia by regulating T-cell function. Interestingly, activation of CD84 promotes the expression level of differentiated genes of M-MDSC and G-MDSC and up-regulates the expression of PD-L1 in MDSC, effects which result in the suppression of T-cell function [24].

**Fig. 1:**
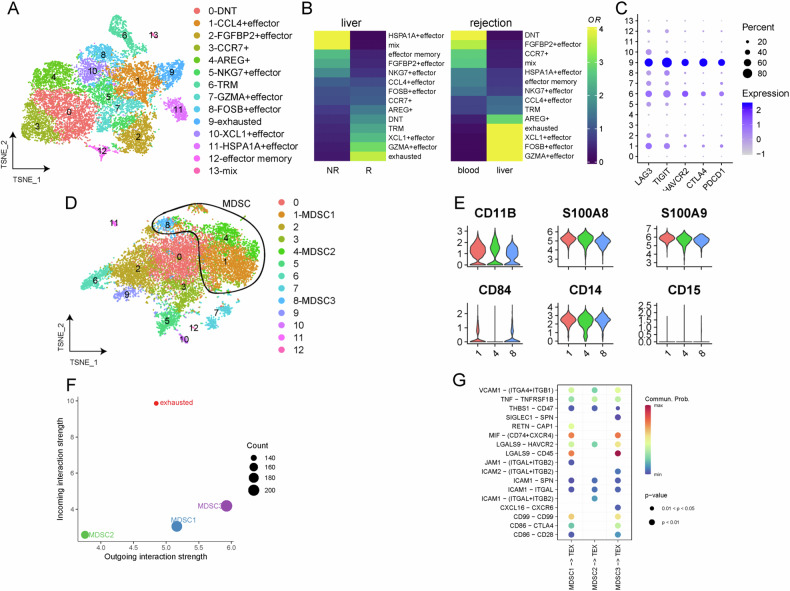
Single-cell sequencing of patients after liver transplantation. **A** T cells were divided into 14 clusters, of which cluster 9 represents exhausted T cells. **B** Left: In liver tissue, exhausted T cells are more likely to be present in rejected samples than in non-rejected samples; Right: in rejected samples, exhausted T cells are more likely to be present in liver samples than in blood. **C** Exhausted marker genes LAG3, TIGIT, HAVCR2, CTLA4 and PDCD1 were highly expressed in cluster 9 cells. **D** Myeloid cells were divided into 13 clusters, of which cluster1, 4 and 8 were MDSCs. **E** The expression levels of CD11b, S100A8, S100A9, CD84, CD14 and CD15 in the 3 clusters of MDSCs. **F** The intercellular communication signal results showed that cluster 1 and 3 MDSCs had stronger output signals, and the exhausted T cells received the strongest signal. **G** The ligand-receptor pairs of 3 groups of MDSCs interacting with exhausted T cells.

Based on published data on single-cell sequencing of patients after LT [4], we re-analyzed and found close interactions between CD84-positive MDSC and exhausted T cells (Fig. 1). So, this study aims to investigate potential changes in the number of bone marrow-derived MDSCs (especially M-MDSCs) in liver grafts in the post-LT state. In addition, we are interested in examining whether infiltrated M-MDSC promotes immunosuppression to alleviate hepatic injury in rejection responses by inducing CD8 + T-cell exhaustion, and whether this immunosuppression function can be regulated by CD84.

## Results

### Phenotypes and distribution of MDSCs after liver transplant

**Fig. 2:**
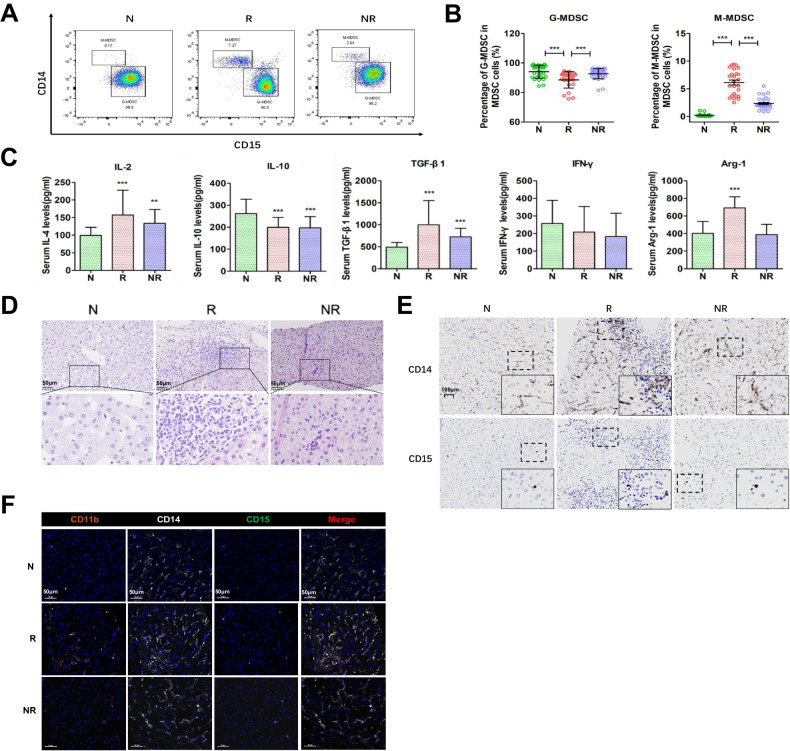
Dynamic changes in phenotypes and distributions of MDSC and cytokines in normal, non-rejected and rejected patients. **A** Proportion of different types of MDSC in PBMC from LT patients. **B** The percentages of M-MDSC and G-MDSC in PBMC from LT patients. **C** Serum cytokine levels (IL-2, IL-10, TGF-β1, IFN-γ and Arg-1) in LT patients. **D** HE staining of liver demonstrating histopathological changes within different groups (200×). **E** Comparisons of infiltration levels of CD14+ and CD15+ cells in liver tissues (200×). **F** The distribution of MDSC in liver tissues was detected by multiple immunofluorescent analysis. Fixed and paraffin-embedded tissue sections were labeled against CD11b (orange), CD15 (green), CD14 (white), and DAPI (blue). Scale bar, 50 μm; N normal, R rejected group, NR non-rejected group; **P < 0.01, ***P < 0.001.

Peripheral blood and liver biopsy tissue samples were collected from post-transplant patients and healthy volunteers to assess potential dynamic changes in MDSCs. Increases in the frequency of MDSCs were observed in LT recipients. Specifically, the percent of M-MDSC was significantly increased, while that of G-MDSC significantly decreased in the rejection versus non-rejection group (*P* < 0.001, Fig. 2A, B). Results of serum cytokine assays revealed that in the LT rejection group, IL-2, TGF-β and Arg-1 were significantly increased (*P* < 0.05) while IL-10 was significantly decreased (*P* < 0.05) as compared with that observed in the non-rejected and normal groups. IFN-γ levels in both the LT rejected and non-rejected groups were lower than that obtained in the normal group (*P* > 0.05, Fig. 2C).

A massive infiltration of immune cells, mainly within the portal area was observed in liver biopsy samples of the LT rejection group. In addition, edema, impairment of hepatic cells, and even partial congestion in hepatic sinuses and apoptosis were observed (Fig. 2D). Immunocytochemistry results indicated that CD14+, as well as CD15+ cells appeared locally assembled, especially in immune cell infiltration areas in the LT rejection group (Fig. 2E). Using multiple immunofluorescent techniques, we further found that both the numbers and rates of CD11b + CD14+ (markers for M-MDSC) and CD11b + CD15+ double-positive cells (markers for G-MDSC) were significantly increased in the LT rejection group, mainly distributed in portal areas or scattered within hepatic lobules (Fig. 2F). These results suggest that there may be a relationship between the infiltration of specific MDSC phenotypes and LT rejection.

### Dynamic changes of MDSCs in the mouse OLT model

**Fig. 3:**
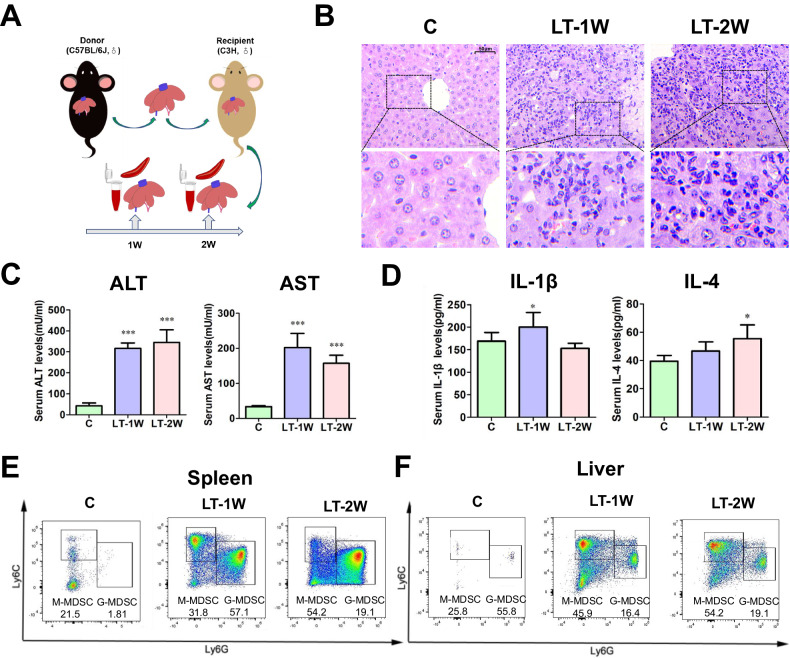
Dynamic changes of MDSC in the mouse liver transplantation model. **A** For the mouse liver transplantation (LT) model, male C57BL/6 J mice served as donors and male C3H mice as recipients. **B** HE staining of liver tissues showed that the infiltrations of immune cells were most significant in LT-1W group (400×). **C** Serum ALT and AST levels at different timepoints post-LT. **D** Serum IL-1β and IL-4 levels at different timepoints post-LT. **E** In spleen, the percentage of M-MDSC peaked at LT-1W and gradually decreased, while that of G-MDSC continued to increase from week 1 to 2 after LT. **F** In liver, the proportion of both M-MDSC and G-MDSC gradually increased from week 1 to 2 after LT determined using flow cytometry. C control group, LT-1W post-transplant 1 week group, LT-2W post-transplant 2 weeks group. *P < 0.05, ***P < 0.001.

The mouse OLT model was employed to provide a method for assessing the dynamic changes of MDSCs that may occur after LT (Fig. 3A). We found that a massive infiltration of lymphocytes, monocytes, macrophages and granulocytes were observed in the hepatic lobular and portal areas of LT-1W mice, which resulted in regional edema, necrosis of hepatic cells and congestion in hepatic sinuses or venules. These impairments were alleviated in the LT-2W group (Fig. 3B). ALT and AST levels were significantly increased after LT (*P* < 0.05). ALT levels gradually increased from week 1 to 2 (*P* < 0.05), while that of AST peaked at week 1, then gradually decreased (*P* < 0.05). As for serum cytokine levels, IL-1β peaked at LT-1W (*P* < 0.05), while IL-4 gradually increased, achieving statistical significance at LT-2W (*P* < 0.05) (Fig. 3C, D). The proportions of overall MDSCs significantly increased in both the liver and spleen of LT mice. G-MDSCs and M-MDSCs were both significantly increased in the liver grafts. In spleen, the percentage of M-MDSC peaked at LT-1W and gradually decreased, while that of G-MDSC continued to increase from week 1 to 2 after LT. Greater changes in the numbers and proportions of M-MDSCs were observed within the intra-hepatic region (Fig. 3E, F).

### Changes in T-cells after liver transplant

**Fig. 4:**
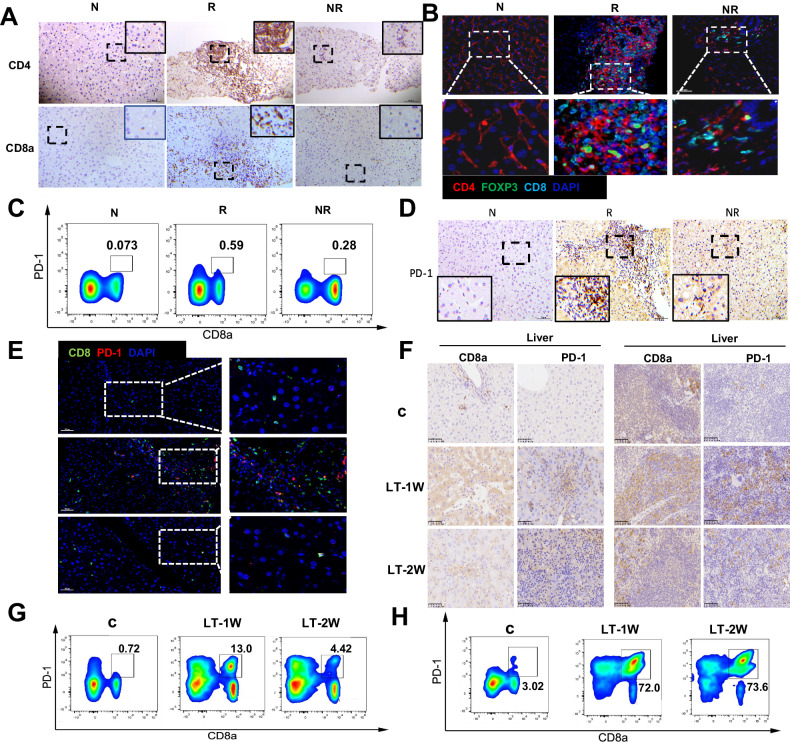
Changes in T cells after liver transplantation. **A** Immunohistochemistry results of infiltrated CD4+ and CD8 + T-cells following LT indicated that massive amounts of T cells were observed in rejected liver tissues (400×). **B** Distribution of CD4+ (red), CD8+ (cyan) and FOXP3+ (green) cells in liver tissues were determined using multiple immunofluorescence (Scale bar, 50 μm). **C** Dynamic changes of PD-1 + CD8 + T cells in PBMC of LT recipients were determined using cytometric analysis. **D** Distributions of PD-1+ cells in liver tissues of LT patients were determined using immunohistochemistry (400×). **E** Expressions and distributions of PD-1+ (red) and CD8+ (green) cells in liver tissues of LT patients were determined using immunofluorescence (Scale bar, 50 μm). **F** Expressions and distributions of PD-1 and CD8+ cells in liver and spleen tissues of the mouse LT model were determined using immunohistochemistry (400×). Dynamic changes of PD-1 + CD8 + T cells in mice spleen (**G**) and liver grafts (**H**) were determined using cytometric analysis. N normal, R rejected group, NR non-rejected group, C control group, LT-1W post-transplant 1 week group, LT-2W post-transplant 2 weeks group.

Then, we investigated the dynamic changes of T cells in LT patients and mouse LT model. Immunohistochemistry results revealed that there were increases in liver-infiltrated CD4+ and CD8+ cells in rejected patients, while the infiltration was markedly less in the non-rejected group, but still greater than that observed in the normal group (Fig. 4A). Multiple immunofluorescent images of liver tissues showed that the number of CD4+ and CD8 + T cells were notably increased in the LT rejection group, with a similar trend observed in FOXP3+ cells. In addition, massive amounts of CD4 and FOXP3 double-positive cells (defined as Treg cells) were observed in the rejection group (Fig. 4B). These results suggest that different phenotypes of lymphocytes participated in the pathological processes associated with LT rejection.

In flow cytometric analysis of PBMC, PD-1 + CD8 + T cells were found increased in post-LT patients, with maximal levels observed in the rejection group (Fig. 4C). Similarly, immunohistochemistry results also showed that maximal increases in PD-1+ cells were present in the rejection versus non-rejection group (Fig. 4D). As shown in Fig. 4E, more intrahepatic PD-1 + CD8+ cells were observed in immune cell-infiltrated areas of liver grafts in the rejection group. To further investigate the expressions of CD8 and PD-1, as related to the liver grafts and spleen at different periods after LT, we assessed samples from the mice LT model. Results of the immunohistochemical assay showed that the number of CD8 + T cells increased after LT and peaked at LT-2W in both liver and spleen. PD-1+ cells in the liver increased gradually after LT, while in the spleen they peaked at LT-1W (Fig. 4F). Flow cytometric analyses of liver and spleen tissue samples showed similar results (Fig. 4G, H). These findings suggest that PD-1 + CD8 + T cells post-LT appear to be associated with LT rejection.

### Interactions between MDSCs and CD8 + T cells related to liver transplant

**Fig. 5:**
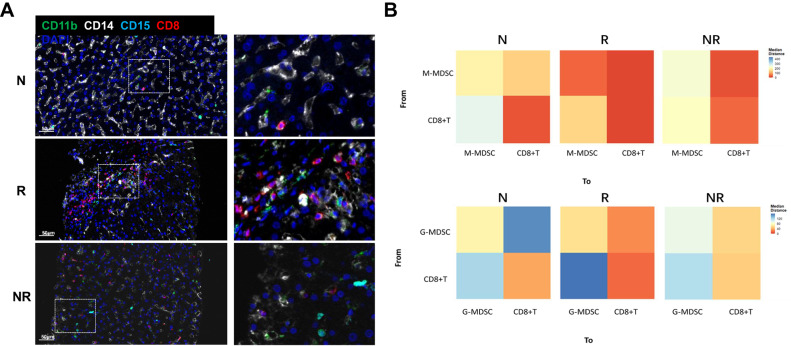
Spatial relationships between CD8 + T cells and different phenotypes of MDSC in different groups. **A** Distribution of MDSC and CD8 + T cells in liver tissues of LT patients were determined using multiple immunofluorescence. Fixed and paraffin-embedded tissue sections were labeled against CD11b (green), CD8 (red), CD15 (cyan), CD14 (white), and DAPI (blue). Scale bar, 50 μm. **B** The spatial relationships between MDSC and CD8 + T cells in different groups were determined based on their labeling (CD11b + CD14+ for M-MDSCs; CD11b + CD15+ for G-MDSCs). N normal, R rejected group, NR non-rejected group.

To investigate the potential spatial relationships between M-MDSCs and CD8 + T cells, a multiple immunofluorescent staining analysis of liver biopsy samples from LT patients was performed. We found that CD11b, CD14 double-positive cells (M-MDSC) and CD11b, CD15 double-positive cells (G-MDSC) demonstrate a substantial degree of contiguity with that of CD8 + T-cells in the LT rejection versus non-rejection group (Fig. 5A). When using different phenotypes of MDSC as a central point along with a 25μm radius range to search for CD8 + T-cells, we found that the spatial distance between both types of MDSCs and CD8 + T-cells were drawn closer after LT. Notably, the closest distance was observed between M-MDSCs or G-MDSCs and CD8 + T cells in the LT rejection group, with this distance increasing as a function of decreases in the severity of the rejection; the result also proved the close relationship was more obvious between CD8 + T cells and M-MDSCs rather than G-MDSCs. (Fig. 5B). Accordingly, it seems that the most intense interaction resides within the immune cell-infiltrated areas of the LT rejection group.

### Relationships between the expressions of CD84 and PD-L1 after liver transplant

**Fig. 6:**
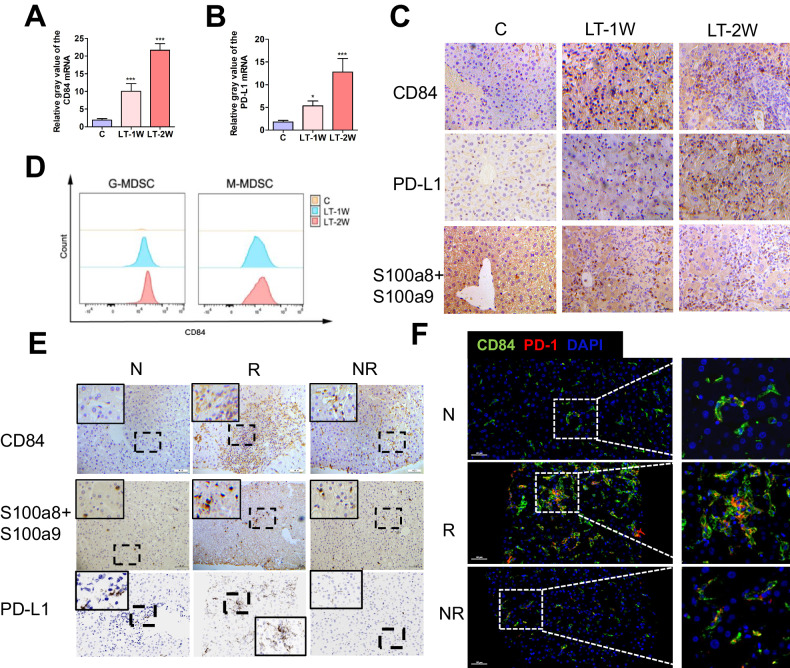
Relationships between CD84 and PD-L1 after liver transplantation. mRNA levels of CD84 (**A**) and PD-L1 (**B**) in liver grafts of mice were determined using qRT-PCR. **C** Expressions and distributions of CD84 + , PD-L1+ and S100a8 + S100a9+ cells in mice liver grafts were determined using immunohistochemistry (400×). **D** Flow cytometry analysis showed the numbers of CD84+ gated in M-MDSC were greater than in G-MDSC in mice liver grafts. **E** Expressions and distributions of CD84 + , S100a8 + S100a9+ and PD-L1+ cells in LT patients were determined using immunohistochemistry (400×). **F** Expressions and distributions of CD84+ and PD-L1+ cells in LT patients were determined using immunofluorescent staining. Fixed and paraffin-embedded liver tissue sections were labeled against CD84 (green), PD-L1 (red) and DAPI (blue). Scale bar, 50 μm. N normal, R rejected group, NR non-rejected group, C control group, LT-1W post-transplant 1 week group, LT-2W post-transplant 2 weeks group, *P < 0.05; ***P < 0.001.

As the ligand of PD-1, PD-L1 is crucial in combining with PD-1 to regulate the function of T-cells. Given that CD84 was reported to promote expression levels of differentiated M-MDSC and G-MDSC genes and up-regulate the expression of PD-L1 in MDSCs, we investigated the relationship between CD84 and PD-L1 expression. In the determination of CD84 mRNA levels in the mice model, it showed a gradual, statistically significant increase from post-LT week 1 to 2 (*P* < 0.05, Fig. 6A). PD-L1 mRNA level was also increased after LT, comparable with that of CD84 (Fig. 6B). The immunohistochemistry results showed a gradual increase trend in CD84 after LT, and the expressions of PD-L1 and S100a8 + S100a9 were also increased from week 1 to 2 (Fig. 6C). It is noteworthy that the numbers of CD84+ gated in M-MDSC were greater than in G-MDSC in mice liver grafts (Fig. 6D). Similarly, in liver samples from LT patients, increased numbers of CD84 +, S100a8 + S100a9+ and PD-L1+ cells were observed after LT, which was particularly prevalent within immune cell infiltrated areas in the LT rejection group (Fig. 6E). Meanwhile, significant increases in the number of CD84 and PD-L1 double-positive cells were present after LT, especially in the LT rejection group (Fig. 6F). These results indicate that the expressions of CD84 and PD-L1 are closely related following LT.

### CD84 regulates M-MDSC function through activating the Akt/Stat3 pathway

**Fig. 7:**
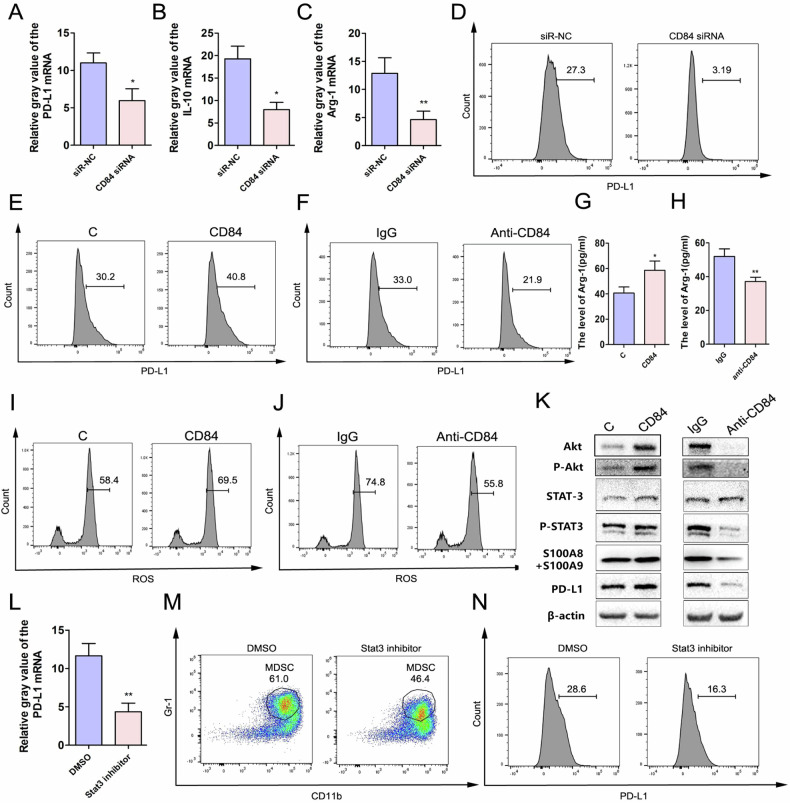
CD84 activates the Stat3 pathway to promote M-MDSC immunosuppression in vitro. Relative mRNA levels of PD-L1 (**A**), IL-10 (**B**) and Arg-1 (**C**) in M-MDSC treated with CD84 siRNA and control group. **D** Flow cytometry analysis showed the expressions of PD-L1 in M-MDSCs after treated with CD84 siRNA. **E** The expressions of PD-L1 in M-MDSCs after treated with CD84 protein. **F** The expressions of PD-L1 in M-MDSCs after treated with anti-CD84 protein; The levels of Arg-1 in supernate of M-MDSC treated with CD84 protein (**G**) and anti-CD84 protein (**H**) were detected by ELISA. Changes in ROS production in M-MDSC treated with CD84 protein (**I**) and anti-CD84 protein (**J**) were determined using flow cytometry. **K** Western blot determinations of the Stat3 pathway (Akt, P-Akt, Stat3, P-Stat3, S100a8 + S100a9 and PD-L1) protein levels in M-MDSC treated with CD84 and anti-CD84 protein. **L** After treatment with the Stat-3 inhibitor, NSC 74859 (MCE, USA), the mRNA levels of PD-L1 in bone marrow-derived and induced cells was decreased; The proportion of MDSC (**M**) and the amount of PD-L1 + MDSC (**N**) were both decreased when treated with the Stat-3 inhibitor than DMSO group. *P < 0.05; **P < 0.01; ***P < 0.001.

When M-MDSCs were transfected with CD84 siRNA in vitro, the relative expressions of mRNA (Fig. 7A, B) and protein levels (Fig. 7c) of PD-L1, IL-10 and Arg-1 were significantly decreased compared to that observed in the control group (siRNA-NC). Results of the flow cytometric assay provided further evidence that lower levels of PD-L1 expression along with the proportions of PD-L1 + MDSCs were present in M-MDSCs transfected with CD84 siRNA (Fig. 7D). We then treated M-MDSCs with CD84 protein or anti-CD84 protein to examine the mRNA levels of PD-L1, IL-10 and Arg-1. The expression levels of Arg-1 were increased in the CD84 protein-treated group (*P* < 0.05), while decreased in the anti-CD84 protein-treated group (*P* < 0.01). Similar effects were observed in flow cytometric analysis, with the expression of PD-L1 and the proportion of PD-L1+MDSCs being increased after treatment with CD84 protein and significantly decreased in the group treated with anti-CD84 protein (Fig. 7E, F). The levels of Arg-1 in cell supernatant showed a similar trend (Fig. 7G, H). The production of ROS and the proportion of ROS-positive cells in M-MDSC treated with CD84 and anti-CD84 protein also showed a similar trend (Fig. 7I, J).

In determination of the Akt/Stat3/PD-L1 pathway, we found that the protein levels of PD-L1 and S100a8 + S100a9, along with Akt, P-Akt, Stat3 and P-Stat3 were significantly decreased in anti-CD84 group, while increased in CD84 protein group compared with IgG treated control groups (Fig. 7K). These results further supported the findings that CD84 activated the Akt/Stat3 pathway to promote the expressions of PD-L1 and S100a8 + S100a9 in M-MDSCs.

To verify the significance of the Stat3 pathway in MDSC, NSC 74859 (MCE, USA, 100 mg/mL dissolved in DMSO), the Stat-3 inhibitor was added. There was a decrease in the proportion of MDSC, PD-L1+ cells, and the mRNA levels of PD-L1, which provides evidence that the activation of Stat3 pathway was crucial for the maturation and functioning of MDSC (Fig. 7L–N).

### CD84 is crucial for M-MDSC-induced exhaustion of CD8 + T cell

**Fig. 8:**
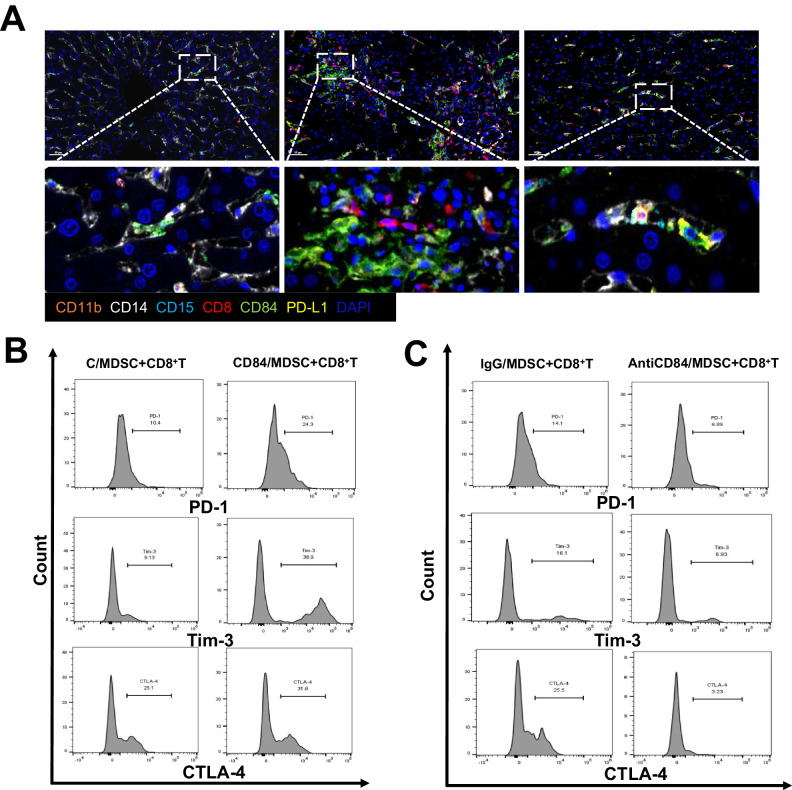
CD84 is crucial for M-MDSC-induced CD8 + T cell exhaustion. **A** Spatial relationships between CD84 + PD-L1+MDSCs and CD8 + T cells were measured by multiple immunofluorescent staining. Fixed and paraffin-embedded liver tissues were labeled against CD11b (orange), CD14 (white), CD15 (cyan), CD8 (red), CD84 (green), PD-L1 (yellow) and DAPI (blue). Scale bar, 50 μm. **B** The flow cytometry analysis revealed the changes of exhaustion markers in CD8 + T cell co-cultured with M-MDSC treated with CD84 protein and control group. **C** The changes of exhaustion markers in CD8 + T cell co-cultured with M-MDSC treated with anti-CD84 protein and control group. N normal, R rejected group, NR non-rejected group, C control group.

As shown in Fig. 8A, the number of CD84 + PD-L1+MDSCs increased significantly after LT. The greatest proportion was found in the LT rejection group, and these cells maintained close spatial contiguity with CD8 + T cells. Such findings suggest that a notable interaction may exist between these two cells.

M-MDSCs treated with CD84 and anti-CD84 protein were further co-cultured with CD8 + T cells in vitro. In the CD84 protein group, increased levels of exhaustion markers PD-1, Tim-3 and CTAL-4 of CD8 + T cells were observed (Fig. 8B). In contrast, treatment with anti-CD84 protein produced an opposite effect (Fig. 8C), which means an impaired suppression function of M-MDSC was present when CD84 was blocked. This further proved our hypothesis that CD84 promotes M-MDSC-induced exhaustion of CD8 + T-cell.

## Discussion

New perspectives on overcoming LT rejection have emerged with recent insights into the understanding of T-cell exhaustion and the immunosuppression function of MDSC. Inducing CD8 + T-cell exhaustion represents a novel approach to inhibit rejection and induce immune tolerance [8], with the target cell of MDSCs being reported to be T-cells. With use of human recombinant HSP90α, it is possible to increase the resistance to apoptosis of normal monocytes, stimulate PD-L1 and IDO-1 expressions through TLR4 signaling, while leaving the production of ROS and nitric oxide (NO) and expressions of Arg-1 and CD73 unchanged. These monocytes can be converted into MDSCs, which then inhibit T cell proliferation [25]. The ability to induce MDSC suppression is mainly achieved through the production and release of cytokines including Arg-1, iNOS, TGF-β, itaconic acid and IL-10, or through intercellular surface protein binding involving such factors as PD-L1/PD-1or CD86/CTLA-4 [26]. Activated Arg-1 is one of the major mediators for MDSCs to exert immunosuppression effects. Arg-1 can convert L-arginine (which is necessary for lymphocytes) into urea and L-ornithine and, in this way, inhibit its proliferation [27]. Simultaneously, MDSCs can also consume the cysteine necessary for T cells and inhibit T cell activation [28].

As a group of heterogeneous cells produced in pathophysiological processes, MDSCs play an important role in immuno-regulation of rejection responses in organ transplantation. Immunosuppression activities of MDSCs have been reported in the areas of kidney and lung transplantation in humans [29, 30], and the mechanisms of MDSC involved with inducing impairments in proliferation and function of pathogenic T-cells, induction of Treg production and infiltration in grafts have been studied in mouse models of skin, heart and corneal transplantation [31–33]. The inhibitory effects of MDSCs on T-cells can be achieved through multiple pathways including the secretion of mediators that reduce T-cell proliferation and cytokine-producing function [34, 35], as well as through inducing T-cells to express exhaustion-related indicators [36, 37]. MDSCs are also important in the immunosuppressive Tumor MicroEnvironment (TME). For example, S100a9 was reported to participate in the TME by promoting chemotaxis and activation of MDSCs through the p38MAPK/TLR4 mediated NF-κB pathway, as regulated by RAGE [38]. Besides, S100a8 + S100a9 is crucial for MDSCs to be fully functional. Under conditions of chronic inflammation, the immunosuppression capacity of MDSC was enhanced by inhibition of its differentiation resulting from TNF-α via S100a8 + S100a9 and its corresponding receptors [39].

CD84 was originally cloned from the human B cell line cDNA library [24], but little work has been directed toward investigating its biological properties on lymphocytes and myeloid. Recent findings have revealed that CD84 is involved in the development and progression of diseases by regulating T-cell function [40]. In Multiple Myeloma (MM), the expression of CD84 in MM cells was found to be decreased, and these cells could secret Macrophage Migration Inhibitory Factor (MIF), which was able to promote CD84 expression. Activated CD84 has been shown to increase the expression of differentiation genes of M-MDSC and G-MDSC and up-regulate PD-L1 expression in MDSCs, both factors can then jointly contribute to the inhibition of T-cell function [41].

Based on the above findings, we hypothesized that M-MDSC, regulated by CD84, suppressed CD8 + T cell by inducing its exhaustion. To prove this hypothesis, we first analyzed PBMC from LT patients. In these patients, CD84 content in both G-MDSC and M-MDSC cells was increased, especially in those with high rejection levels. S100a8 + S100a9 positive cells were concentrated in immune cell-infiltrated areas of rejected liver grafts. Compared with that of the control group, the number of CD4 + T and CD8 + T cells were increased in PBMC of LT patients, while the proportions of these cells in CD3 + T cells varied with the rejection state. The proportion of PD-1 + CD8 + T cells in LT patients was also significantly increased, especially in the rejection group. PD-1 is an important exhaustion indicator of T cells. After prolonged exposure to antigens, immune responses of T cells gradually wane and may eventually result in a functional impairment [42]. These impaired T cells fail to differentiate into memory T cells and subsequently evolve into exhausted T cells [43]. We found that a large number of PD-1+ exhausted T cells were present in PBMC from LT patients, which indicates that some of these CD8 + T cells have become “exhausted”. We further propose that spatial distance between different types of cells may serve as the basis of interactions, which is supported by the results of multiple immunofluorescent assays. Specifically, the closest distance was found between M-MDSCs and CD8 + T cells, which would enhance the potential interaction between these two cells. Notably, this contiguity was particularly prevalent in the LT rejection group.

As the samples obtained from patients were limited in both time points and quantity, and it was not possible to acquire spleen samples, the mouse LT model was included to examine the changes and relationships between PD-L1, CD84 and S100a8 + S100a9 in both liver grafts and spleen. The expression levels of CD84 and S100a8 + S100a9 were significantly increased in the rejected liver. For PD-L1, we observed a significant increase at 2 weeks after surgery. The trend was similar in the mouse LT model. Expression levels of CD84 and PD-L1, as well as the double-positive cells, increased after LT in mice. The above results suggest that both CD84 and PD-L1 are involved in the pathophysiological processes of immune rejection after LT.

To further verify whether CD84 regulates M-MDSC to express PD-L1, we investigated the relationships with an in vitro model. We first knocked down the expression levels of CD84 in M-MDSCs using CD84 siRNA. With this treatment, the relative mRNA expression levels of PD-L1, IL-10 and Arg-1 in M-MDSC were significantly decreased, and the proportion of PD-L1+ in M-MDSCs was also decreased. In contrast, M-MDSCs treated with CD84 protein showed increased mRNA levels of PD-L1, IL-10, TGF-β and Arg-1, and the proportion of PD-L1 + M-MDSCs was also increased. Meanwhile, there was also an increase in the production of ROS and the proportion of ROS+ cells under these conditions. ROS participates in signal transduction pathways by regulating cellular metabolism and oxidative stress, thus regulating a number of biological activities [44]. As an important medium for MDSC to function, ROS not only activates anti-oxidant pathways, but also induces transcriptional programs related to MDSC function. Under pathological conditions, T-cell function can be inhibited by the ROS released from MDSC [45]. With the change in activity of CD84 in MDSCs, the expression levels of PD-L1 and S100a8 + S100a9 show a corresponding change, suggesting that there may be some connection among these three factors. In Myelodysplastic Syndrome (MDS), the expression of PD-1 in hematopoietic stem/progenitor cells and PD-L1 in MDSC are increased, and c-Myc is necessary for S100a9 to up-regulate PD-1/PD-L1 expression [46]. As mentioned in the introduction part, Stat3 pathway was crucial to the function of MDSCs. Recent studies have proved CD84 up-regulated PD-L1 expression in MDSCs by Akt/mTOR pathway [41]. Meanwhile, another research showed Akt was able to activate Stat3 pathway by suppressing salt-inducible kinase 1 (SIK1), which is an inhibitor of Stat3 pathway by binding to Stat3 [47]. Based on these studies, we assume that CD84 activates the Stat-3 pathway by activating Akt. In our in vitro model, after treatment with CD84 protein, the Akt/Stat-3 pathway in MDSC was activated, with the expression levels of PD-L1 and S100a8 + S100a9 increased. Stat3/Arg-1 signaling was reported to play an important role in the expansion and activation of M-MDSC cells in patients with Ankylosing Spondylitis (AS). AS-associated M-MDSC shows high levels of P-Stat-3, and inhibition of Stat-3 activity decreases Arg-1 activity thus diminishing its immunosuppression function [48]. Napaubucasin, the Stat-3 inhibitor, completely depletes immunosuppression function as demonstrated in both mice and humans, and improves the survival rate within melanoma bearing mice [16]. Silencing Stat-3 expression or suppressing Stat-3 activity has been shown to decrease PD-L1 expression in NK/T lymphoma cell lines [49]. P-Stat-3 not only binds to the PD-L1 promoter, but also forms P-Stat1-Stat3 dimerization with P-Stat-1 within the cytoplasm, which can then be transferred to the nucleus to bend the PD-L1 promoter and induce PD-L1 expression [50]. In this study, we found that after blocking CD84 in M-MDSCs, both ROS production in M-MDSCs and the proportion of ROS + M-MDSCs decreased significantly.

The above results provide robust evidence that CD84 mediates PD-L1 expression in M-MDSCs by regulating phosphorylation levels of Stat-3, and influences the production of ROS. Based on these, we further co-cultured M-MDSCs with CD8 + T cells and treated with either CD84 or anti-CD84 protein. T-cell exhaustion was then evaluated by the levels of PD-1, CTLA-4 and Tim-3. Increases in these indicators have been reported to represent impaired T-cell function and exhaustion [51, 52]. In the group where M-MDSCs were treated with CD84 protein, co-cultured CD8 + T-cells expressed increased levels of PD-1, Tim-3 and CTLA-4, whereas the trend was reversed in M-MDSCs blocked by anti-CD84 protein. These demonstrate that alteration in CD84 activity result in changes of M-MDSC function and induction of CD8 + T-cell exhaustion.

## Conclusions

In conclusion, CD84 promotes M-MDSC-induced CD8 + T-cell exhaustion and dysfunction by increasing the expression levels of PD-L1, thus alleviating the rejection after LT. These findings improve our understanding of the role MDSC plays in LT rejection and immune tolerance, and provide a potential strategy for treating rejection.

## Subjects and methods

### Clinical samples

Data from 60 LT patients admitted to the Liver Transplant Center of Beijing Friendship Hospital, Capital Medical University for regular evaluation and 33 healthy volunteers serving as controls were collected. All of the LT patients underwent an ultrasonic-guided liver biopsy for pathological assessment. They received immunosuppressive therapy consisting of tacrolimus (FK506), mycophenolate mofetil and glucocorticoids, with drug doses adjusted based on serum concentrations. Baseline data collected included ABO blood type, graft type, ischemia time, protopathy and blood biochemistry consisting of alanine aminotransferase (ALT), aspartate aminotransferase (AST) and total bilirubin (T-Bil) levels.

The degree of rejection was evaluated by the Banff Score as generated from two blinded, experienced pathologists. Liver with a Rejection Active Index (RAI) < 3 was defined as non-rejected while RAI of 3–9 was defined as rejected. Based on this criteria, 25 LT patients were allocated to the rejection group and 35 to the non-rejection group. This study was approved by the ethics committee of Beijing Friendship Hospital, Capital Medical University, and informed consent was signed by each patient and healthy volunteer.

### Mouse orthotopic LT model

To establish the mouse LT model, SPF grade male C57BL/6 mice aged 8–10 weeks served as donors and C3H mice aged 8–10 weeks as recipients (purchased from the Sibeifu Biotechnology Co., Ltd, Beijing). The selection of donor and recipient mice was random, and the surgical procedure employed consisted of the established Kamada’s ‘two-cuff’ method. The recipient mice were euthanized at either 1 week (LT-1W) or 2 weeks (LT-2W) post-transplant. If jaundice was observed within 1 week after LT, mice were excluded. HE staining was performed with the left lateral lobes of the grafts. The animal experiment was approved by the animal ethics committee of Beijing Friendship Hospital, Capital Medical University.

#### Flow cytometry analysis

FACS analysis was performed on Attune™ NxT (Thermo, America) and the data were analyzed using Flowjo software (v10.8.1).

Antibodies used included (purchased from Biolegend, USA): anti-human-CD11b (PE/Cyanine7), anti-human-CD14 (APC/Cyanine7), anti-human-LA-DI-DA (FITC), anti-human-CD15 (PerCP/Cy5.5), anti-human-CD84 (PE), anti-human-CD127/IL-7Rα (APC), anti-human-CD279/PD-1 (PE), anti-human-CD25 (BV421), anti-human-CD4 (Alexa Fluor® 700), anti-human-CD3 (FITC), anti-human/mouse-CD11b (FITC), anti-mouse-CD45 (APC/Cyanine7), anti-mouse-CD84 (PE), anti-mouse-Ly-6C (APC), anti-mouse-Ly-6G (BV421), anti-mouse-Ly-6G/Ly-6C (Gr-1)(APC), anti-mouse-F4/80 (AlexaFluor®700), anti-mouse-CD274 (PD-L1)(PE/Cy7), anti-mouse-CD3 (PerCP/Cyanine5.5), anti-mouse-CD4 (FITC), anti-mouse-CD8a (Alexa Fluor® 700), anti-mouse-CD279/PD-1 (PE), and anti-mouse-Tim-3 (BV711). Dead cells were stained with Zombie Aqua (BV510).

#### Western blotting

Proteins were extracted from liver grafts of recipient mice and cell lysates. The following antibodies were used: anti-Stat3, anti-p-Stat3, anti-β-actin (CST, America), anti-PD-1, anti-PD-L1, anti-CD84 and anti-S100a8 + S100a9 (Abcam, UK). All primary antibodies were diluted to 1:3000 with primary antibody diluent (Abcam, UK). Membranes were then incubated with horseradish peroxidase-conjugated goat anti-rabbit polyclonal secondary antibodies. Protein bands were visualized using the Bio-Rad gel imaging system (USA).

#### Immunofluorescence and immunohistochemistry

Paraffin-embedded liver (human and mice) and spleen (mice) tissues were incubated with primary antibodies: CD14, CD15, CD4, CD8, PD-1, PD-L1 and FOXP3. For immunohistochemistry, the primary incubation was followed by incubation with a secondary antibody, while for immunofluorescence, the fluorescent secondary antibody mixture and the anti-fluorescent attenuation tablet containing DAPI was added. Results were then assessed using an Olympus laser scanning confocal microscope (Japan).

#### Quantitative reverse transcription-PCR (RT-qPCR)

Total RNA was isolated from mouse livers or MDSC using the TRIzol reagent, and reverse-transcribed into cDNA templates using a PrimeScriptTM RT reagent kit. The primers used in qPCR included:

IFN-γ-F:ATGAACGCTACACACTGCATC; IFN-γ-R:CCATCCTTTTGCCAGTTCCTC;

β-actin-F:GGCTGTATTCCCCTCCATCG; β-actin-R: CCAGTTGGTAACAATGCCATGT;

IL-10-F:GCTCTTACTGACTGGCATGAG; IL-10-R:CGCAGCTCTAGGAGCATGTG;

TGF-β1-F:CTCCCGTGGCTTCTAGTGC; TGF-β1-R:GCCTTAGTTTGGACAGGATCTG;

Arg-1-F:GGTCTCTCACGTCATACTCTGTTTC; Arg-1-R: ATCGGAGCGCCTTTCTCAAAA;

PD-L1-F:CTCGCTTCGGCAGCACA; PD-L1-R:AACGCTTCACGAATTTGCGT;

CD84-F:CCTGGT-CATTAGAGACCTGAGG; CD84-R:TGCTGCCATAACTTACGGTAG；

The 2−△△CT method was used for the quantitative analyses of all samples.

#### Cell isolation and sorting

MDSCs were isolated from the bone marrow of male C57BL/6 mice and then cultured with RPMI-1640 medium, GM-CSF (20 ng/mL, Peprotech, USA) and IL-6 (20 ng/mL, Peprotech, USA) for 3–6 days. The MDSC Kit (Miltenyi, Germany) was used for the sorting of M-MDSC. The MS separator was placed within a magnetic separator containing an appropriate MACSS® magnetic field as required for sorting (Mini & MidiMACS™, MS separator, Miltenyi, Germany).

CD8 + T cells were isolated from the spleens of male C57BL/6 mice using the mouse CD8a+T Kit (Miltenyi, Germany). The LS separator was placed within a magnetic separator containing an appropriate MAC magnetic field as required for sorting (Mini & MidiMACS™, LS separator, Miltenyi, Germany). Sorted cells were assessed by flow cytometry analysis.

#### SiRNA transfection in vitro

CD84 siRNA was purchased from RiboBio Co., Ltd. Guangzhou China, and verified by qRT-PCR. The siRNA sequence was CAAGATCTACTACCTTCAT. Lipo 3000 (Lipofectamine 3000 Kit, Invitrogen, USA) method was used to transfect, and the CD84 siRNA concentration was adjusted to 100 nM.

#### Datasets availability

The single-cell sequencing datasets presented in this study can be found in online repositories. The name of the repository and accession number can be found below: NGDC Genome Sequence Archive (https://ngde.cncb.ac.cngsa-human); HRA002091.

#### Statistical analysis

Data are presented as means ± SEMs. Statistical comparisons among the three groups were performed by one-way ANOVA or Kruskal-Wallis test with post hoc Tukey test for pairwise comparison of subgroups. Student’s *t*-tests were used for comparisons of two groups. Statistical analysis was performed using GraphPad version 9.0 (GraphPad Software). A two-sided *P* < 0.05 was considered statistically significant.

### Supplementary information

ORIGINAL WB results

ORIGINAL qPCR results

Supplement Materials

Checklist

## Data Availability

All data generated or analyzed during this study are available in this article.
