# Supplementary figures and images for: Myeloid-derived suppressor cells-induced exhaustion of CD8 + T-cell participates in rejection after liver transplantation
Source: Cell Death Dis. 2024 Jul 16;15(7):507. doi: 10.1038/s41419-024-06834-z (PMC11252260; doi:10.1038/s41419-024-06834-z)

C      CD84    IgG    anti-CD84

stat3

88 KDa

p-stat3

88 KDa

S100a8/9

13 kDa

PD-L1

50 kDa

$\beta$ -actin

42 kDa

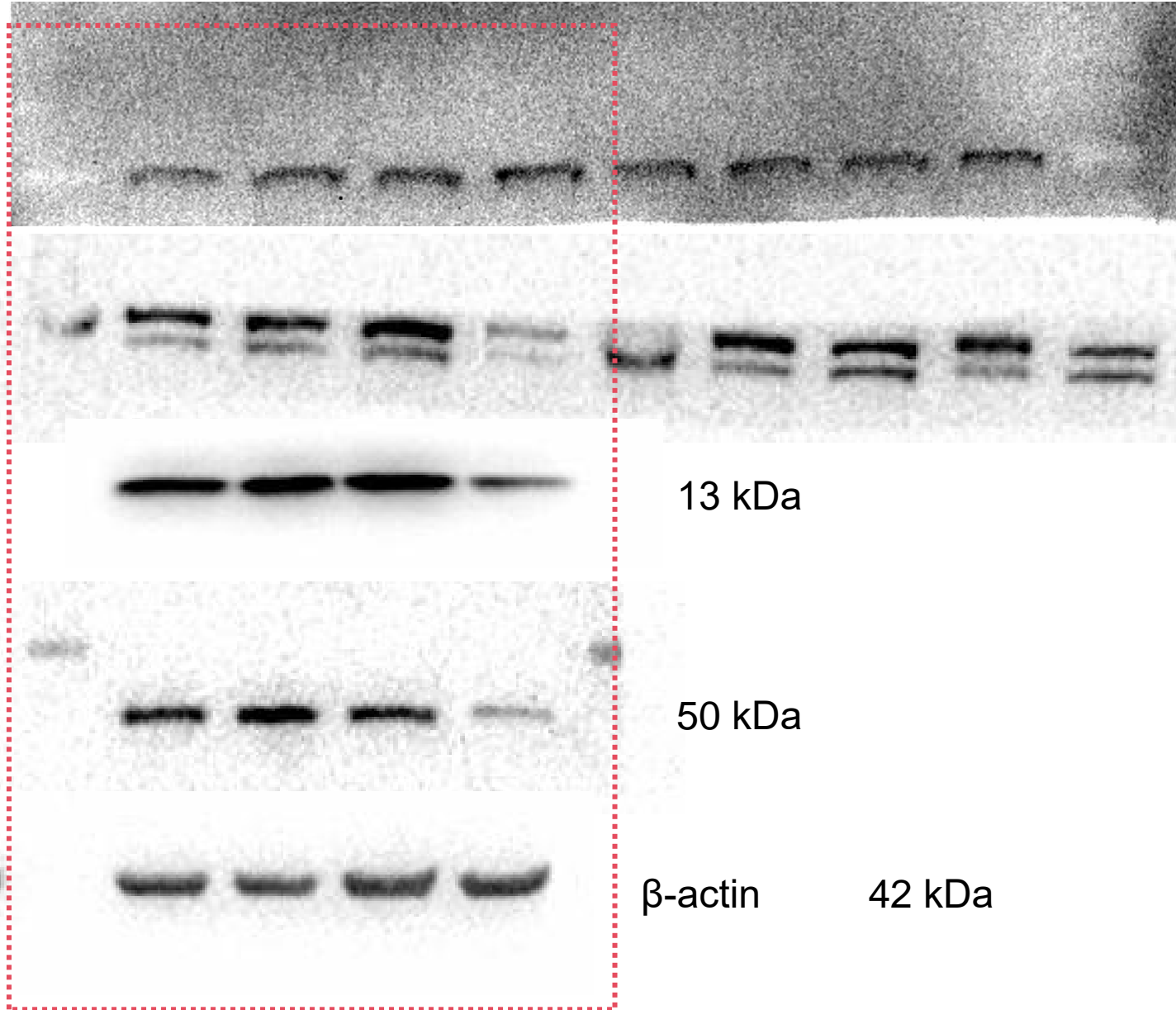

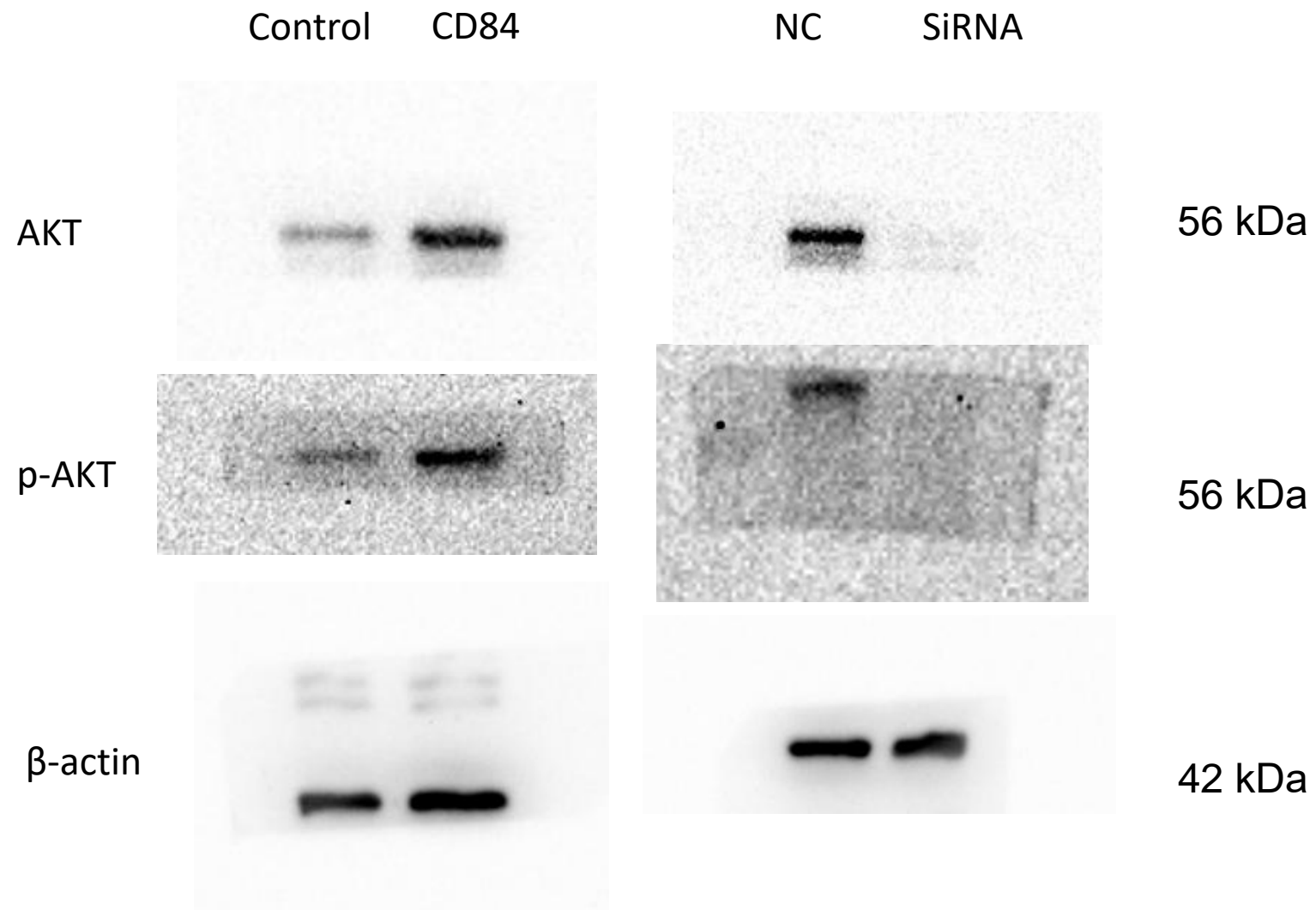

Supplement: Supplementary file 1 — ORIGINAL WB results [file 41419_2024_6834_MOESM1_ESM.pdf]
