# Supplementary material for: Myeloid-derived suppressor cells-induced exhaustion of CD8 + T-cell participates in rejection after liver transplantation
Source: Cell Death Dis. 2024 Jul 16;15(7):507. doi: 10.1038/s41419-024-06834-z (PMC11252260; doi:10.1038/s41419-024-06834-z)
Supplement: Supplementary file 3 — Supplement Materials [file 41419_2024_6834_MOESM3_ESM.pdf]

Antibody	Fluorescein	Vendor	Reactivity	Catalog number
Zombie Aqua™ Fixable Viability Kit	BV510	Biolegend	Human	423101
CD127(IL-7Rα)	APC	Biolegend	Human	351342
CD279(PD-1)	PE	Biolegend	Human	379210
CD25	BV421	Biolegend	Human	302630
CD3	FITC	Biolegend	Human	317306
CD45	BV421	Biolegend	Human	304032
CD15	PerCP/Cy 5.5	Biolegend	Human	301922
CD14	APC/Cy 7	Biolegend	Human	325620
CD11b	PE/Cy 7	Biolegend	Human	301322
HLA-DR	FITC	Biolegend	Human	327006
CD84	PE	Biolegend	Human	326008
CD4	Alexa Fluor 700	Biolegend	Human	344622
CD8a	BV605	Biolegend	Human	344742
CD11b	FITC	Biolegend	Human/Mouse	101206
CD8a	Alexa Fluor 700	Biolegend	Mouse	100730
CD4	FITC	Biolegend	Mouse	100406
CD3	PerCP/Cy 5.5	Biolegend	Mouse	100218
Ly-6C	APC	Biolegend	Mouse	128016
Ly-6G	BV421	Biolegend	Mouse	127627
Ly-6G/Ly6C(Gr-1)	APC	Biolegend	Mouse	108411
CD45	APC/Cy 7	Biolegend	Mouse	157204
CD84	PE	Biolegend	Mouse	122806
F4/80	Alexa Fluor 700	Biolegend	Mouse	123130
CD279(PD-1)	PE	Biolegend	Mouse	114117
CD366(Tim-3)	BV711	Biolegend	Mouse	134021
CD274(PD-L1)	PE/Cy 7	Biolegend	Mouse	124314

Supplement Column 1: The detailed information of Flow cytometry antibodies used.

Primary Antibody	Vendor	Reactivity	Host	catalog number
CD14	Abcam	Human	Rabbit	ab183322
CD15	Abcam	Human	Rabbit	ab115993
CD8	Abcam	Human/Mouse	Rabbit	ab316778
CD4	Abcam	Human/Mouse	Rabbit	ab288724
FOXP3	Abcam	Human/Mouse	Rabbit	ab215206
PD-1	Abcam	Human	Rabbit	ab206378
PD-L1	Abcam	Human	Rabbit	ab228415
S100a8+S100a9	Abcam	Human/Mouse	Rabbit	ab288715
CD84	Abcam	Human	Rabbit	ab131256
Stat3	CST	Mouse	Rabbit	#9139
p-Stat3	CST	Mouse	Rabbit	#9145
β-actin	CST	Human/Mouse/Rat	Rabbit	#4967
Akt(pan)	CST	Human/Mouse/Rat	Rabbit	#4691
p-Akt	CST	Human/Mouse/Rat	Rabbit	#4060
PD-L1	Abcam	Mouse	Rabbit	ab213480
PD-1	Abcam	Mouse	Rabbit	ab214421

Supplement Column 2: The detailed information of primary antibodies used.
